# Alterations of serum biomarkers associated with lung ventilation function impairment in coal Workers: A cross-sectional study

**DOI:** 10.1186/1476-069X-10-83

**Published:** 2011-09-27

**Authors:** Jimin Zou, Xianming du Prel Carroll, Xianhong Liang, Dongmei Wang, Chao Li, Baojun Yuan, Sandra Leeper-Woodford

**Affiliations:** 1 Department of Clinical Laboratory, Attached Kai Luan Hospital of North China Coal Medical College, Tangshan, 063000, China; 2 Department of Community Medicine, Mercer University School of Medicine, 1550 College Street, Macon, GA 31207, USA; 3 Department of Neurology, Beijing Tiantan Hospital, Capital Medical University, No. 6 Tiantan Xili, Beijing, 100050, China; 4 Division of Basic Medical Sciences, Mercer University School of Medicine, Macon, 1550 College Street, Macon, GA 31207, USA; 5 Department of Social Medicine and Health Education, School of Public Health, Nanjing Medical University, Nanjing 210029, China

**Keywords:** Coal workers' pneumoconiosis, pulmonary function, serum biomarkers, interleukins

## Abstract

**Background:**

Previous studies have demonstrated that alterations in certain circulating biomarkers may be correlated with Coal workers' pneumoconiosis (CWP). This study investigated the relationship between changes of serum biomarkers and pulmonary function during the development of CWP.

**Methods:**

Lung function parameters and specific serum indices were measured in 69 non-smoking coal workers, including 34 miners with CWP, 24 asymptomatic miners and 11 miners with minimal symptoms. The associations between changes in pulmonary function and serum indices were tested with Pearson's correlation coefficients. Multivariable analysis was used to estimate the predictive power of potential determinant variables for lung function.

**Results:**

Compared to healthy miners, lung function (FVC, FEV_1_, FEF_50_, FEF_75_, FEF_25-75 _% of predicted values) was decreased in miners with CWP (*p *< 0.05). Increased serum matrix metalloproteinase-9 (MMP-9) was associated with decreased FVC% of predicted values in the asymptomatic miners (*r *= -0.503, *p *= 0.014).

**Conclusions:**

In coal mine workers, alterations of lung function parameters are associated with the development of CWP and with changes in circulating MMP-9, TIMP-9, IL-13 and IL-18R. These serum biomarkers may likely reflect the pathogenesis and progression of CWP in coal workers, and may provide for the importance of serum indicators in the early diagnosis of lung function injury in coal miners.

## Background

Despite implementation of rules and regulations for safer limits to dust exposure in the workplace, Coal workers' pneumoconiosis (CWP) is still an occupational health problem [1,2]. Coal miners with pneumoconiosis often experience symptoms such as chronic cough, sputum production and episodes of wheezing, which suggests the presence of airway dysfunction. Ventilatory defect is a common finding among miners with CWP [3].

Although pathophysiological mechanisms have not been elucidated, it has been proposed that following exposure to coal dust, lung tissue responds subsequently in a series of three steps: activation and accumulation of inflammatory cells [4]; activation and proliferation of fibroblasts [5]; and enhanced accumulation of mesenchymal cells and production of collagen [6]. Each of these steps involves a complex interplay of diverse cell types and mediators, including cytokines and other biomarkers that have been implicated as participants in these processes.

It has been previously reported that interleukin-1beta (IL-1β), IL-8, IL-18 and monocyte chemoattractant protein-1 (MCP-1) are important in recruiting and activating leukocytes, and also in pro-inflammatory cytokine production [7-10]. The Th_2 _cytokine IL-13 may be involved in inflammation and remodeling, which accompanies lung fibrosis [11], while the antifibrotic effects of IL-9 may be associated with a limitation of the type 2 polarization observed in pulmonary fibrosis [12].

Similarly, intercellular adhesion molecule-1 (ICAM-1), CD40, and CD40 ligand (CD40L) have been documented in localized tissue inflammation and fibrotic responses [13-15]. Matrix metalloproteinases (MMPs), which are initiators of the enzymatic degradation process of extracellular matrix, are critical in the tissue remodeling process [16,17]. Elevated levels of MMPs have been noted during early stages of pulmonary silica exposure [18].

Studies of pulmonary fibrosis-sensitive mice have shown positive correlations between tissue and serum cytokine levels, suggesting that blood indices may be used as surrogate markers for tissue cytokines [19]. Previous studies from our laboratory have demonstrated that changes in serum IL-18, IL-18 receptor (IL-18R), MCP-1, sICAM-1, MMP-9, and tissue inhibitors of metalloproteinase-9 (TIMP-9) may be involved in the pathogenesis of CWP [20-24], but little is known about the association between these serum biomarkers and lung function impairment.

We hypothesized that the alterations in serum biomarkers were related to pulmonary function impairment in CWP. To test this possibility, spirometry parameters of expiratory flow lung capacity FVC, FEV_1_, FEF_25_, FEF_50_, FEF_75_, FEF_25-75 _and FEV_1_/FVC were determined as clinical evidence of respiratory injury [25]. The associations between these lung function parameters and levels of serum biomarkers (IL-1β, IL-8, IL-9, IL-13, IL-18, IL-18R, MCP-1, sICAM-1, MMP-9, TIMP-9, sCD40, and sCD40L) were analyzed in non-smoking coal workers with CWP and two matched control groups, asymptomatic miners and miners with minimal symptoms (group 0+).

## Methods

### Subjects

Coal miners were physically examined and history of clinical conditions and smoking habits were assessed at the Kai Luan Prevention and Treatment Institute of Occupational Disease from March 1 to December 30 of 2008. Those study subjects selected were all non-smoking males working under similar mine environmental conditions, having normal liver and kidney function with no coronary artery disease, hypertension, diabetes mellitus or autoimmune disease. None of the subjects reported any recent or current infection.

A posteroanterior chest radiograph was taken on each subject. Two experienced physicians read the radiological appearances following the diagnostic criteria of pneumoconiosis GBZ 70-2002 [26], and three Categories of the lung condition were determined as follows: Category 1, few small opacities present, distribution to at least two areas of lung; Category 2, numerous small opacities present, distribution to more than four areas of lung; Category 3, a large opacity present, not less than 20 mm × 10 mm in size. Asymptomatic miners were those subjects who had chest radiographs without evidence of pneumoconiosis. Group 0^+ ^miners were those subjects who had chest radiographs with only borderline evidence of pneumoconiosis-like opacities that were not definitive enough to be diagnosed as pneumoconiosis category 1.

According to the diagnostic criteria, 40 miners with CWP category 1 and 20 miners from group 0+ were recruited; 25 matched asymptomatic miners were also selected randomly for the study. Excluding missing data due to inappropriate cooperation in the lung function test, and insufficient or haemolysis samples, 69 subjects completed the study. Theses study subjects included 34 miners with CWP stage 1, 11 group 0+ miners, and 24 asymptomatic miners.

This study was approved by the Ethics Committee of the Attached Kai Luan Hospital of North China Coal Medical College. Informed consent was obtained from all study subjects.

### Pulmonary ventilation function tests

Spirometry tests were performed by trained personnel utilizing an automatic pulmonary functions testing system, the Masterscreen PFT (JAEGER, Germany). Lung ventilation function parameters were measured from each subject in this study. These parameters included the percent predicted values for forced vital capacity (FVC), forced expiratory volume in 1 second (FEV_1_), forced expiratory flow after 25% of vital capacity has been expelled (FEF_25_), forced expiratory flow after 50% of vital capacity has been expelled (FEF_50_), forced expiratory flow after 75% of vital capacity has been expelled (FEF_75_), forced expiratory flow from 25% to 75% of vital capacity (FEF_25-75_), and the percentage of FEV_1 _to FVC (FEV_1_/FVC).

### Serum biomarkers analysis

Serum concentrations of IL-1β, IL-8, IL-13, IL-9, IL18, IL-18R, MCP-1, sICAM-1, MMP-9, TIMP-9, sCD40, and sCD40L in all of the coal miner subjects were determined according to the manufacturer's instructions using commercially available ELISA kits (R&D Systems). The concentrations of sera indices were calculated by comparing the values measured with a standard curve constructed using known concentrations of the corresponding indices.

### Statistical analysis

All data were analyzed with the SPSS16 software. Lung ventilation function parameters, except for the FEV_1_/FVC ratio, were expressed as percentages of predicted values (% p). The lung function parameters were of normal distribution; the differences among groups (mean ± SD) were examined with ANOVA and the Tukey-Kramer multiple comparisons tests.

Serum IL-1β, IL-8, IL-13, IL-18R, sICAM-1, TIMP-9 and sCD40L were of normal distribution. Logarithm transformations were performed on MMP-9, MCP-1, IL-9, IL18 and sCD40 before statistical analysis.

Pearson's correlation coefficients were calculated to examine the strength of association between the lung function parameters and the levels of serum factors. Multiple stepwise analyses were used to estimate the predictive power of potential determinants for lung function in different groups respectively; p < 0.05 was considered as statistically significant.

## Results

The demographic characteristics of the three groups of the coal miners are presented in Table 1. In order to minimize the selection bias, we compared the distribution of characteristics, including age and dust-exposed time, and there were no significant differences among the three groups.

**Table 1:** Demographic Characteristics of CWP and Control Groups

Subjects	Numbers	Age (yr, Mean ± SD)	Exposure duration (yr, Mean ± SD)
CWP	34	58.2 ± 5.7	26.6 ± 5.4

Group 0+	11	57.0 ± 5.0	28.5 ± 7.4

Asymptomatic miners	24	57.8 ± 5.9	27.5 ± 6.8

### Comparisons of lung ventilation functional parameters among CWP and control groups

**Figure 1:**
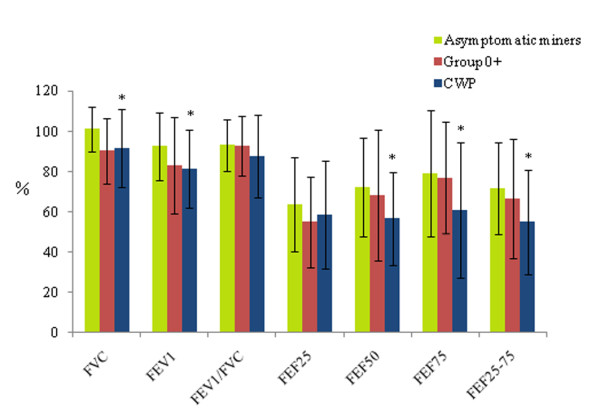
**Comparisons of Lung Ventilation Functional Parameters among Patients with CWP and two Control Groups**. The percent predicted values of FVC, FEV_1_, FEF_50_, FEF_75_, and FEF_25-75 _in patients with CWP were significantly decreased when compared with the respective parameters in asymptomatic miners: compared with asymptomatic miners, *p *< 0.05.

As shown in Figure 1, when compared with the data from asymptomatic miners, most of the lung ventilation functional parameters were in a decreasing trend in patients with CWP. The FVC, FEV_1_, FEF_50_, FEF_75 _and FEF_25-75 _values were significantly decreased (*p *= 0.033, 0.035, 0.024, 0.037, and 0.017, respectively), while FEV_1/_FVC and FEF_25 _showed no significant differences. When the same parameters of group 0+ were compared with asymptomatic miners, the values were in a decreasing trend but not significant (Figure 1). Except for FVC and FEF_25_, the FEV_1_/FVC, FEF_50_, FEF_75_, FEF_25-75 _and FEV_1 _parameters showed decreasing trends in patients with CWP when compared with group 0+, but there was also no significant difference (Figure 1).

### Correlations of lung ventilation functional parameters and levels of serum biomarkers in asymptomatic miners

The results of Pearson's correlation analysis indicated that serum levels of IL-18 in the asymptomatic miners showed a trend towards positive association with all lung ventilation functional parameters; on the other hand, levels of IL-18R, sCD40, sCD40L, MMP-9, TIMP-9 and sICAM-1 showed a trend towards inverse association with all lung ventilation function parameters. Besides these findings, levels of MCP-1, IL-1β, IL-8, IL-13 and IL-9 did not show any relationship with lung ventilation functional parameters in asymptomatic miners.

With respect to MMP-9 in the asymptomatic group, decreases of FVC values were correlated with the increased levels of MMP-9 in these miners (*r *= -0.503, *p *= 0.014). In addition, as shown in Table 2 the results of multiple stepwise analyses indicated that the decrease of FVC values was significantly associated with the increased MMP-9 values in the asymptomatic miners.

**Table 2:** Multiple Stepwise Analysis of Dependent Variable by Lung Ventilation Functional Parameters and Independent Variable by Serum Biomarker Levels in Asymptomatic Miners

Dependent Variable	Independent Variable	Unstandardized Coefficients	Standardized Coefficients	t	*p *Value
FVC (% p)	MMP-9	-0.154	-0.057	-2.677	0.014

Significance: *p *< 0.05

### Correlations of lung ventilation functional parameters and serum biomarker levels in group 0+ miners

The results of Pearson's correlation analysis showed that in the group 0+ subjects, the increased serum TIMP-9 levels were significantly correlated with increased FEF_25 _values in these miners (*r *= 0.710, *p *= 0.014). Serum levels of IL-13 and MMP-9 showed inverse associations with all ventilation function parameters, and increased IL-13 levels were significantly correlated with decreased FEV_1_/FVC and FEF_25 _values (*r *= -0.765, *p *= 0.010; *r *= -0.672, *p *= 0.033, respectively); increased MMP-9 levels were also significantly associated with decreased FVC values (*r *= -0.620, *p *= 0.042).

The results of multiple regression analysis as shown in Table 3 indicate that the decreases of FVC values in these same subjects were significantly associated with increases of serum MMP-9 values (*p *= 0.04); decreases of FEV_1_/FVC were significantly associated with increases of IL-13 values (*p *= 0.01); and decreases of FEF_25 _values were associated with decreases of serum levels of TIMP-9 (*p *= 0.028). Furthermore, the results of multiple stepwise analyses also indicated that serum TIMP-9 had the strongest correlation and serum MMP-9 had the weakest correlation with the lung function parameters in group 0+ miners.

**Table 3:** Multiple Stepwise Analysis of Dependent Variable by Lung Ventilation Functional Parameters and Independent Variable by Serum Biomarker Levels in Group 0+

Dependent Variable	Independent Variable	Unstandardized Coefficients	Standardized Coefficients	*t*	*p *Value
FVC (% p)	MMP-9	-0.098	-0.065	-2.449	0.040

FEV_1_/FVC (%)	IL-13	-1.439	-0.428	-3.364	0.010

FEF25 (% p)	TIMP-9	0.184	0.687	2.675	0.028

Significance: *p *< 0.05

In addition, with regards to the results of Pearson's correlation analysis, although serum levels of TIMP-9 and sCD40L in the group 0+ miners showed a trend towards positive association with all ventilation functional parameters, the serum levels of MCP-1, IL-1β, IL-8, IL-18, IL-18R, IL-9, sCD40, sICAM-1 did not show any trends of association with lung ventilation functional parameters in this group.

### Correlations of lung ventilation functional parameters and serum biomarker levels in group CWP miners

**Figure 2:**
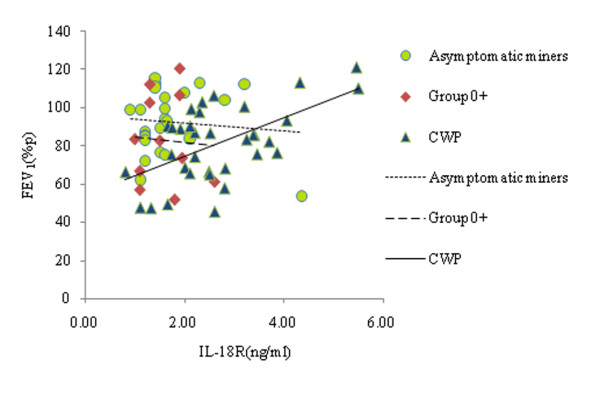
**Correlations of FEV_1 _and Serum Levels of IL-18R in the Coal Workers**. Scatter plot showed that decreased serum IL-18R levels in the CWP subjects correlated with decreased FEV_1 _(r = 0.570, *p *< 0.0005) in patients with CWP. Serum levels of IL-18R showed an inverse trend association with FEV_1 _in asymptomatic miners and group 0+ miners.

The results of Pearson's correlation analysis indicated that serum levels of IL-18R in the CWP miners showed trends towards positive associations with all ventilation function parameters, and also enhancements in IL-18R levels in the subjects with CWP were correlated with increased FEV_1 _(Figure 2), FEF_50_, FEF_75 _and FEF_25-75 _values (*r *= 0.570, *p *< 0.0005; *r *= 0.406, *p *= 0.017; *r *= 0.392, *p *= 0.022; *r *= 0.475, *p *= 0.004, respectively). However, multiple stepwise analyses of the same data suggest that decreases of FEV_1 _were significantly associated with decreases of serum IL-18R (Table 4). Other lung ventilation function test parameters did not show significant correlations with IL-18R levels in these subjects.

**Table 4:** Multiple Stepwise Analysis of Dependent Variable by Lung Ventilation Functional Parameters and Independent Variable by Levels of Serum Biomarkers in CWP Group

Dependent Variable	Independent Variable	Unstandardized Coefficients	Standardized Coefficients	*t*	*p *Value
FEV_1 _(% p)	IL-18R	11.044	0.736	3.437	0.006

Significance: *p *< 0.05

In addition, the results indicated that the levels of serum sCD_40_L in the CWP group showed trends towards inverse association with all lung ventilation function parameters; however there was no significant correlation. Serum levels of MCP-1, IL-1β, IL-8, IL-18, IL-13, IL-9, MMP-9, TIMP-9, sCD40, and sICAM-1 in the CWP miners did not show trends in relation to ventilation functional parameters.

## Discussion

This study demonstrated that the lung ventilation function parameters, the percent predicted values for FVC, FEV_1_, FEF_50_, FEF_75 _and FEF_25-75_, showed significant decreases in patients with CWP when compared with the respective values in the asymptomatic miners group. These results were similar to those observed in previous studies [3]. Longitudinal studies in coal miners have shown that exposure to coal mine dust contributes to a decline in FEV_1 _[27,28]. Mamuya et. al. also reported that exposure to coal mine dust was associated with airway limitation as measured by FEV_1_/FVC and the predicted FEV_1_(%) [29]. By comparing the three groups of miners in the present study, our lung ventilation function data provide further evidence that airway dysfunction may be a predominant feature in the initial development and progression of the lung disease associated with coal mining.

The present study also revealed that the alterations of lung function parameters may be associated with the changes in concentration of certain serum biomarkers in these coal miner subjects.

MMP-9 is produced constitutively by neutrophils and eosinophils [30,31]. It has been shown that coal dust particles may stimulate macrophages and neutrophils, and that MMP-9 may be released immediately when these cells are exposed to coal dust [32]. If levels of MMP-9 were continually stimulated to increase, MMP-9 could induce inflammation by activating IL-1β, an auto-inducible cytokine central to the inflammatory reaction [33]. These responses could participate in aberrant remodeling processes which damage the extra cellular matrix and may result in lung injury. Data from this study suggest that MMP-9 may play a role in these processes in coal mine workers and is consistent with the proposition that MMP-9 participates in the development of airway inflammation and pulmonary fibrosis in these miners [34].

A previous study from our laboratory demonstrated that levels of serum MMP-9 in asymptomatic miners were significantly lower when compared with the levels in healthy control subjects who had no coal dust exposure [22]. Also, the levels of serum MMP-9 in group 0+ were elevated slightly when compared with the dust-exposed control group [22]. This previous data supports the results of the present study indicating that increases of serum MMP-9 are related to the impairment of ventilation function, while decreases of MMP-9 levels are correlated with better lung function parameters. These results provide evidence of the correlation between levels of MMP-9 and the progression of lung disease in miners, and may serve as possible reasons why most asymptomatic coal miners in our study did not develop pneumoconiosis.

The results of this study also revealed that the decrease of serum TIMP-9 levels was correlated with decreased ventilation function parameters in the coal miners with minimal symptoms. These findings are the opposite of our previous study which demonstrated that both TIMP-9 and MMP-9 levels in minimally symptomatic miners were elevated slightly when compared with these levels in dust-exposed control miners [22]. These results indicated that TIMPs could be involved in the compensatory responses to the increased serum MMP levels in order to silence the MMP activity [35]. The present data provides evidence that MMP-9 may be involved in the contribution of the impairment of ventilation function in coal miners. The balance between MMP and TIMP activities in a particular microenvironment seems to determine whether physiological homeostatic extra cellular matrix remodeling or excessive proteolysis and possible pathological consequences occur [36]. Therefore, observations on the differences in serum MMP-9 and TIMP-9 levels in group 0+ and healthy miners might indicate that these serum biomarkers may play roles in the progression of lung disease, and may provide for the importance of serum indicators for early diagnosis of lung function injury in coal miners.

Previous studies have demonstrated that the Th_2 _cytokine IL-13 is a powerful in vivo regulator of tissue remodeling with the ability to activate a variety of MMPs and cathepsins, generating destructive and fibrotic structural responses in tissues [37]. Increased IL-13 production has been documented in a variety of diseases characterized by inflammation and remodeling [38-40]. Data presented in our studies showed that the increased concentrations of serum IL-13 were correlated with decreased FEV_1_/FVC and FEF_25 _levels in group 0+ miners. These results are in agreement with an experimental lung fibrosis study which confirmed that the constitutive and/or inducible transgenic overexpression of IL-13 in the murine lung induces airway inflammation involving macrophages, lymphocytes and eosinophils, airway remodeling with subepithelial fibrosis, parenchymal fibrosis, mucus metaplasia, and striking increases in alveolar size, lung size, and pulmonary compliance [37]. The present data demonstrates that increased circulating IL-13 may be associated with ventilation function impairment, which supports our theory that IL-13 plays a role in the pathogenesis of lung inflammatory and alveolar remodeling in pneumonoconiosis and provides evidence that Th2 cell polarization involving IL-13 may favor the development of pulmonary fibrosis [12,14,37].

The data of our study shows that the serum levels of IL-18R are positively associated with changes in FEV_1 _levels in the patients with CWP, suggesting a relationship between levels of IL-18R and ventilation function impairment in these miners. These results were most consistent with an experimental model in which IL-18R was upregulated during differentiation along the Th1 pathway while IL-18R was downregulated during Th2 differentiation [41]. We also found that IL-18R levels had a slight inverse trend with all of the alterations in lung ventilation function parameters in asymptomatic miners. These results provide evidence that the changes of serum IL-18R levels may concomitantly participate in the occurrence and development of CWP in miners.

It was previously reported that IL-18 and IL-18R may be involved in the pathogenesis of pulmonary fibrosis [42]. Other studies have shown that IL-18 serum levels have a trend to inversely correlate with peak expiratory flow [43]. Results of the present study did not show a significant relationship between IL-18 levels and ventilation function parameters, except in asymptomatic miners. These results may indicate that IL-18 is involved in lung function alteration indirectly, perhaps via influencing from Th_1 _and Th_2 _cell responses or other integrated mechanisms [41,44].

This study also revealed that serum levels of both sCD40 and sCD40L showed trends towards inverse correlations with all ventilation functional parameters in the asymptomatic group and miners with CWP. Also, there were inverse correlations between the sCD40L levels and lung function in the CWP group, and positive correlations between sCD40L and lung function alterations in group 0+. If there had been more participants in this study these associations could possibly have been more significant. From our data analysis, we propose that the CD40:CD40L pathway may be involved in the perpetuation of inflammatory, wound healing responses and fibrosis formation, and that these changes might be activated by infiltrating T lymphocytes that facilitate both cellular and humoral immune responses in the lungs of the miners [45].

Our study also indicated that concentrations of sICAM-1 in serum were inversely correlated with all of the lung ventilation function parameters in the asymptomatic miners. This data implies that there may be a correlation between sICAM-1 and lung function impairment. The sICAM-1 may be elaborated via a mechanism involving ICAM-1 inducing inflammatory responses involving monocyte migration and neutrophil influx in the lungs of the miners [46].

With respect to the cytokines IL-8, IL-9, IL-1β and MCP-1, which are known to be important in initiation and maintenance of inflammatory processes and pulmonary fibrosis [7,8,45], the levels of these biomarkers showed no correlations with changes in lung ventilation function parameters in the three groups of miners in our studies. This suggests that these serum biomarkers may have no direct correlation with ventilation function impairment in these coal mine workers.

## Conclusions

The data in this study provide evidence that the alterations of lung ventilation functional parameters in non-smoking coal workers are associated with changes in serum MMP-9, TIMP-9, IL-13, and IL-18R levels. These circulatory biomarkers may play roles in the progression of the development of pulmonary function impairments in coal miners. The levels of MMP-9, TIMP-9 and IL-13 may be monitored as possible markers to estimate or predict lung function changes in minimally symptomatic coal miners. In addition, our study supports suggestions that intervention against changes of MMP-9, TIMP-9, IL-13 and IL-18R levels are warranted to facilitate diminished risk of pulmonary injury in coal workers.

## Abbreviations

CWP: Coal workers pneumoconiosis; Group 0+: miners with minimal symptoms who could not be diagnosed as pneumoconiosis; FVC: forced vital capacity; FEV_1_: forced expiratory volume in 1 second; FEV_1_/FVC: the percentage of the ratio of FEV_1 _and FVC; FEF_25_: forced expiratory flow at vital capacity of 25%; FEF_50_: forced expiratory flow at vital capacity of 50%; FEF_75_: forced expiratory flow at vital capacity of 75%; FEF_25-75_: the forced expiratory flow from 25% to 75% of vital capacity; ELISA: enzyme-linked immunosorbent assay; IL: interleukin; IL-18R: interleukin 18 receptor; sCD40: soluble CD40; sCD40L: soluble CD40 ligand; MMP-9: matrix metalloproteinase 9; TIMP-9: tissue inhibitor of metalloproteinase 9; sICAM-1: soluble intercellular adhesion molecule-1; MCP-1: monocyte chemoattractant protein-1.

## Competing interests

The authors declare that they have no competing interests.

## Authors' contributions

JZ designed and performed experiments, analyzed the results and wrote the manuscript. XPC helped with the interpretation, participating in writing the manuscript and revising it critically for submission. XL assisted with data interpretation and manuscript preparation. DW and CL helped with experiments and manuscript preparation. BY provided funding and materials, and was involved in the experimental design, providing overview of all the steps of experiments. SLW assisted in drafting the manuscript and critically revising its contents. All authors read and approved the final manuscript.
